# Treatment of Severe Obstructive Sleep Apnea Using Mandibular Advancement Device: A Case Report

**DOI:** 10.7759/cureus.39547

**Published:** 2023-05-26

**Authors:** Suliman Alsaeed

**Affiliations:** 1 Preventive Dental Sciences, College of Dentistry, King Saud bin Abdulaziz University for Health Sciences, Riyadh, SAU; 2 Research Center, King Abdullah International Medical Research Center, Riyadh, SAU; 3 Dental Center, Ministry of the National Guard - Health Affairs, Riyadh, SAU

**Keywords:** snoring, oral appliance, orthodontics, obstructive sleep apnea, mandibular advancement devices

## Abstract

Obstructive Sleep Apnea (OSA) is one of the most common sleep disorders. A mandibular advancement device (MAD) can be used to manage primary snoring and OSA cases. It is mostly indicated in cases with mild to moderate OSA. This case reports shows the successful management of severe OSA using MAD. A 34-year-old male presented to the orthodontic clinic with a diagnosis of severe OSA, apnea-hypopnea index (AHI) of 71 events per hour with symptoms of loud snoring, witnessed gasping, morning headache, and excessive daytime sleepiness. The case was managed using MAD to position the lower jaw in a forward position during sleep with 7 mm advancement. Progress sleep study results showed that the AHI reached normal levels, with only two hypopnea events per hour and a complete resolution of apnea episodes. The patient’s symptoms subsided after using MADs. This case report documents that severe OSA can be managed with MAD in suitable cases.

## Introduction

Sleep is one of the most complicated processes that we undergo every day. Human beings are expected to spend one-third of their lives sleeping; this reflects the importance of sleep [1]. It is expected that individuals not only get enough sleep but also get good sleep quality [2]. However, poor sleep quality is not uncommon [3]. There are more than 70 sleep disorders listed in the International Classification of Sleep Disorders (ICSD) [4]. The most common sleep disorder is insomnia followed by obstructive sleep apnea (OSA) [3]. OSA is characterized by intermittent closure of the upper airway that results in oxygen desaturation [5]. The prevalence of OSA ranges from 9% to 38%, and varies between genders and age groups, with high prevalence in males and elderly patients [6]. The most common cause of OSA in adults is obesity [5]. There are other factors that contribute to OSA such as retrognathia of the lower jaw and poor muscular tone [5]. Sleep disorder is diagnosed by polysomnography (PSG) [5]. This diagnostic modality requires that patients are admitted to a sleep disorder center to undergo a complete sleep study. PSG data provides an overall evaluation of sleep quality and reports different physiological measures such as oxygen saturation and airway flow.

OSA can be categorized into three levels based on the apnea-hypopnea index [7,8]. For adults, if the AHI is more than 5 events per hour and less than 15, it is considered mild. Moderate OSA is 15-30 events per hour, while severe OSA is when the AHI is more than 30 per hour [7,8]. There is no maximum limit of AHI in severe cases. The sleep study data can be observed in different sleep stages such as rapid eye movement (REM) or non-REM stages. Also, it is important to evaluate the position of the body in sleep assessment as the supine position is more associated with OSA [8]. 

The main treatment option for OSA is continuous positive airway pressure (CPAP) [5]. It works by keeping the airway open through continuous airway pressure [9]. This treatment modality has a high efficacy; however, the compliance rate of CPAP is poor due to reported discomfort complaints with CPAP, such as mouth dryness, chest discomfort, difficulty in nasal breathing, and inconvenience in wearing [9-11]. An alternative option for the treatment of OSA is oral appliances (OAs), such as mandibular advancement devices (MADs) [12]. These devices work by keeping the lower jaw in a forward position during sleep, which results in open airway passage to decrease snoring and apnea events [12]. However, this option is mainly presented to patients with primary snoring, mild to moderate OSA, or patients with severe OSA but intolerant to CPAP, as these devices have a better compliance rate than CPAP [12].

This case report describes a case of severe OSA that was managed by MAD, and resulted in complete improvement of OSA symptoms and normal PSG variables including AHI.

## Case presentation

A 34-year-old male presented to the orthodontic clinic with a chief complaint of “I need a mouthpiece to treat my snoring and sleep apnea”. The patient was referred from the sleep disorders center with a diagnosis of OSA. The patient’s height was 190 cm, weight of 99 kg, and BMI was 27.4 kg/m^2^. His main symptoms were snoring, morning headache, excessive daytime sleepiness, unrefreshed sleep, fatigue, and low mood throughout the day. The patient had a complete overnight sleep study, polysomnography (PSG), interpreted by a board-certified sleep physician. His AHI was 71 per hour, with a REM AHI of 109 per hour (Table 1). All apnea events were obstructive in nature with no central apnea events. During the sleep study, a CPAP with a pressure of 8 cmH2O was used. The AHI dropped to two events per hour using CPAP (Table 1). However, the patient was intolerant to CPAP. Alternative options were presented to the patient by the sleep physician including MAD and surgical options such as uvulopalatopharyngoplasty (UPPP) and maxillo-mandibular advancement (MMA). The patient preferred the option of non-surgical MAD.

**Table 1:** Polysomnography data PSG: Polysomnography; CPAP: Continuous Positive Airway Pressure; MAD: Mandibular Advancement Device; AHI: Apnea/Hypopnea Index; REM: Rapid Eye Movement; BMI: Body Mass Index

Variables	Baseline PSG	On CPAP	On MAD
AHI	71/hr	2/hr	2/hr
REM AHI	109/hr	6.5/hr	1/hr
BMI (Kg/m^2^)	27.4	27.4	29.9

**Figure 1:**
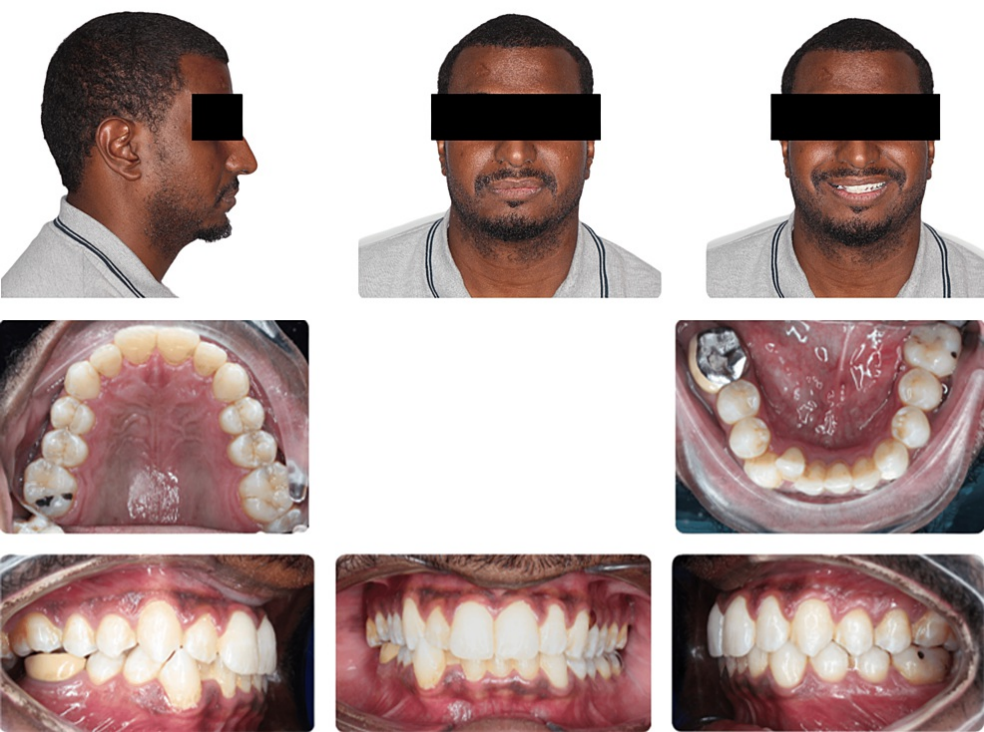
Extra- and Intra-oral photographs

**Figure 2:**
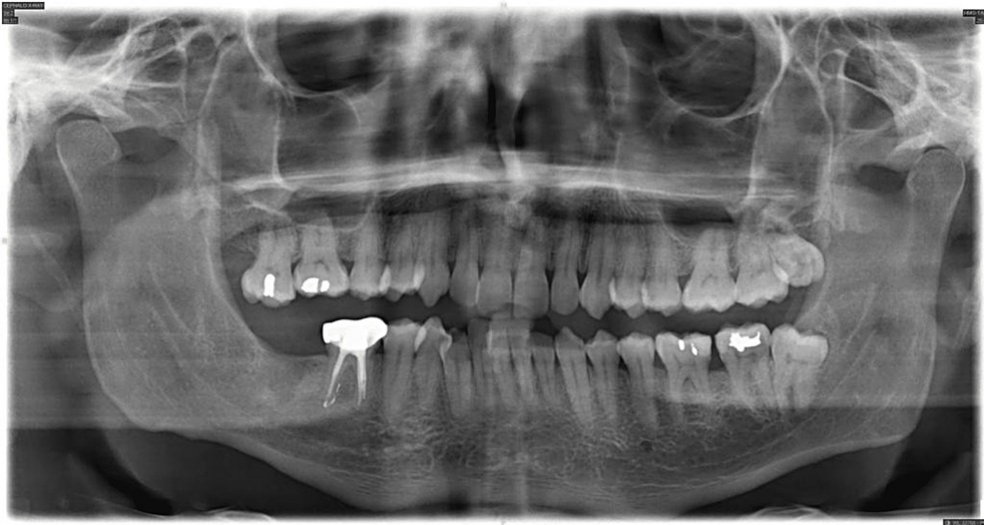
Panoramic radiograph

Orthodontic records were taken, and a complete orthodontic examination was conducted. Extra oral assessment showed a straight profile with a short chin-to-throat length (Figure 1). Upper and lower lips were retruded to the esthetic line. There was a mild facial asymmetry with mandible shifted to the right side. Smile arc was non-consonant and dental display was decreased. The neck circumference was increased. Intra-orally the patient had a class I molar relationship on the left side, while the right side was in class III relationship (Figure 1). Both canines were in class I relationship. Anterior crossbite was present between teeth #12 and #43. The lower midline was shifted to the right side. There was no functional shift. Overbite was relatively deep with 70% overlap of the lower incisors. Lower teeth were severely crowded. Tooth #46 was missing and tooth #17 was over-erupted. There were indentations on the sides of the tongue, reflecting relative macroglossia. The protrusion range of the mandible was 12 mm. The panoramic radiograph showed upper posterior crowding at the upper left third molar area,missing tooth #47 and impaction of the lower left third molar (Figure 2). 

After discussing the orthodontic findings, the patient request was to manage the OSA and to postpone the treatment for his malocclusion. To achieve this goal, a mandibular advancement device was planned. The chosen design was elastomeric MAD (EMA). Upper and lower intra-oral scans were taken, and digital models were printed to fabricate EMA. The starting protrusion position of the mandible was set to be at 60% of the protrusion range, using a 17 mm blue elastic strap.

**Figure 3:**
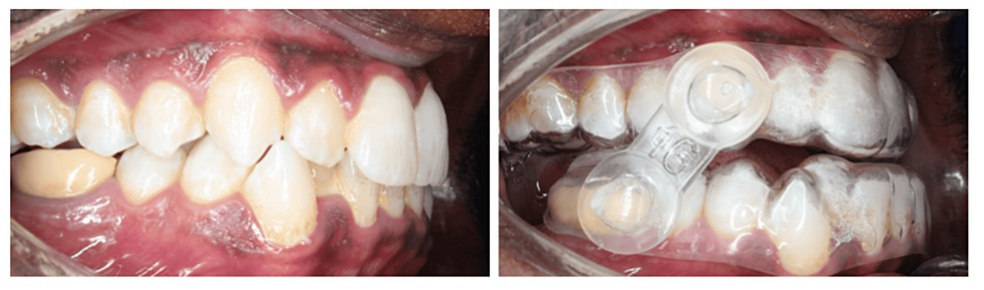
The elastomeric mandibular advancement device used to position the mandible in a forward position.

**Figure 4:**
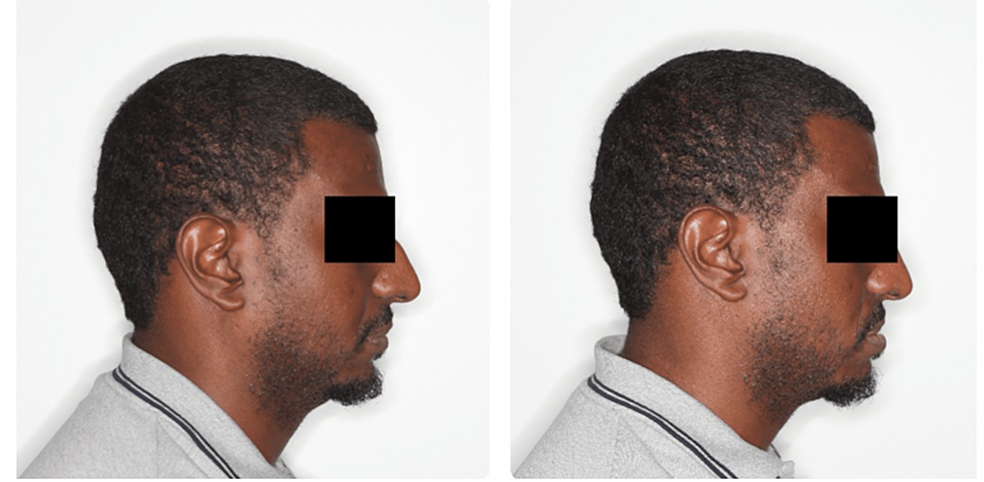
The normal and protruded position of the mandible with the EMA device EMA: Elastomeric Mandibular Advancement

After one week of appliance insertion, the patient reported significant improvement using the oral device at 60% advancement; however, there were remaining snoring events as witnessed by his bed partner. Further advancement was done using a 16 mm clear strap (Figure 3). This helped to hold the mandible further forward using a firm clear elastic strap (Figure 4). After three weeks, the reported OSA symptoms disappeared using the device in this position. There were no more snoring sounds or daytime symptoms such as morning headache, excessive daytime sleepiness, fatigue, or low mood. The patient was referred to his sleep physician for a follow-up PSG to confirm the improvement in sleep quality.

Although the patient’s weight increased from 99 kg to 108 kg, the follow-up PSG showed complete resolution of apnea events with few remaining hypopnea events that were within the normal levels of AHI (normal AHI: 0-5 events per hour). The patient’s AHI dropped from 71/hour to 2/hour with similar efficacy as the 8 cmH2O CPAP that was initiated during the baseline sleep study (Table 1). REM-AHI dropped from 109/hour to 1/hour (Table 1). This sleep study has confirmed, with objective measures, the successful use of MAD to manage a severe OSA case. The patient was scheduled for a yearly follow-up to observe for any side effects, and to fabricate a new appliance if needed.

## Discussion

The use of OAs to treat OSA was documented in 1902 [13]. The American Academy of Sleep Medicine (AASM) published its first official position statement on OAs in 1995 [14]. Since then, the use of OAs became common and further studies were conducted to evaluate its effectiveness and advantages. In 2015, updated guidelines for the use of OAs were published by the AASM [12]. The guidelines state that sleep physicians are recommended to consider OAs for adult patients with OSA who are intolerant of CPAP therapy or prefer an alternative therapy, rather than not treating them at all. The AASM considers this recommendation as a standard of care for OSA patients [12].

In this case, we presented a 34-year-old male patient with severe OSA who was intolerant to CPAP and was referred to the orthodontic clinic to fabricate an OA to treat his OSA. The AASM guidelines suggest that qualified dentists with experience in the field of Dental Sleep Medicine (DSM) use a custom and titratable device to position the mandible forward during sleep, rather than ready-made or non-titratable devices [12]. Furthermore, for evaluation purposes, the AASM guidelines state that a follow-up sleep study is recommended to evaluate the treatment efficacy of OAs [12]. The AASM guidelines were followed in this case from the treatment planning stage to the treatment progress stage and the evaluation stage.

There are different designs for MADs. However, all devices use the same concept of advancing the position of the lower jaw during sleep and keeping the mandible in a forward position to open the airway [15]. There are two main points that need to be considered when designing MADs. First, the appliance should be custom-made. This is essential for the success of MAD treatment as custom devices have a higher effectiveness than ready-made ones and usually have fewer side effects [16]. Furthermore, the device should be titratable. In other words, the device needs to be adjustable to allow for more protrusion of the mandible, if needed. It is common that oral appliance needs to be adjusted in terms of antero-posterior or vertical positions during treatment. If the appliance is not titratable and OSA symptoms persist, the appliance will need to be re-made with a more forward position. This will result in significant additional costs that could have been avoided and easily managed if the appliance was titratable.

A periodic follow-up is needed to ensure treatment effectiveness and to observe for possible side effects. The main side effects of the MADs are proclination of the lower incisors, retroclination of the upper incisors, and posterior open bite [17]. These occlusal changes are common during treatment. However, there are suggested approaches to decrease the rate of these side effects including the use of a morning repositioning splint (MRS). These splints are usually recommended to be worn by the patient for 15-30 minutes in the morning after waking up. Although there are few studies on the effectiveness of MRS, dentists should schedule their patients for periodic follow-up appointments even if MRS is worn by the patient. There are other side effects of MADs including jaw muscle discomfort and sore teeth [12]. These side effects are transient and decrease with time [12]. Also, a yearly follow-up is needed to evaluate the need to fabricate a replacement appliance, especially in case of appliance breakage or significant restorative work that might result in an ill-fitting appliance [12].

The success of OA varies in the literature. The definition of success of OA means that AHI drops to normal levels, below five events per hour [3]. Partial success is when the AHI with OA drops more than 50% but AHI is still more than 5 [3]. Failure is when the AHI reduction with OA is less than 50% [3]. Most studies agreed that as the severity of the OSA increases, the success rate of OA decreases [3,18]. Hence, this reflects the significance of this case report where our patient presented with severe OSA symptoms and AHI of 70 events per hour, which increased to 109 events per hour during REM sleep.

There are different factors that contributed to the successful management of severe OSA in this case. First, it is important to locate the position of obstruction of the airway. Although this can be identified by interventions such as drug-induced sleep endoscopy (DISE), the lack of suitable resources was a barrier to performing this diagnostic evaluation. However, a basic ENT assessment by an ENT specialist indicated that there is no nasal obstruction that is contributing to the snoring or OSA for this patient. This was a positive indication of potentially successful MADs for the management of OSA for this patient. The second factor was that our patient had a facially retruded mandible with relative macroglossia. This has reflected that the area of obstruction could be oral and hence might reflect a predictable successful MAD. The third factor was the amount of protrusion range; our patient has 12-13 mm of protrusion range that allowed for forward positioning of the mandible during sleep. However, the amount of advancement was just 7-8 mm. Other factors for success, in this case, include the use of custom and titratable devices, treatment by qualified orthodontists in the field of DSM, periodic follow-ups, a relatively young patient, and a low BMI [3,12].

Although this report documents a significant success for severe OSA cases, the findings of this case report should be taken with caution as case reports are ranked at the bottom of scientific evidence to draw conclusions. Another limitation of the study is the lack of a DISE report for this case to identify the area of airway obstruction. It would be helpful to evaluate the amount of airway opening with and without the appliance. This was not performed due to the lack of resources. Further studies are needed in the field of severe OSA management using MADs.

## Conclusions

Orthodontists can provide a significant service to OSA patients by involving the field of DSM to deliver optimum care for their OSA patients. For severe OSA cases with skeletally retruded mandible, sufficient protrusion range, CPAP intolerance, and no nasal obstruction, MAD can be a potentially good option rather than no treatment at all. A follow-up PSG is essential to evaluate the improvement of sleep parameters using MADs.

The findings of this case report should be taken with caution as case reports are ranked at the bottom of scientific evidence to draw conclusions. Further research on the potential success of MADs in the treatment of severe OSA is needed, specifically on the predictive factors of MADs' success.
